# PhenoMultiOmics: an enzymatic reaction inferred multi-omics network visualization web server

**DOI:** 10.1093/bioinformatics/btae623

**Published:** 2024-10-17

**Authors:** Yuying Shi, Botao Xu, Zhe Wang, Qitao Chen, Jie Chai, Cheng Wang

**Affiliations:** Department of Biostatistics, School of Public Health, Cheeloo College of Medicine, Shandong University, Jinan 250012, China; National Institute of Health Data Science of China, Shandong University, Jinan 250000, China; National Science Library (Chengdu), Chinese Academy of Sciences, Chengdu 610299, China; Department of Gastrointestinal Surgery, Shandong Cancer Hospital and Institute, Shandong First Medical University and Shandong Academy of Medical Science, Jinan 250000, China; Department of Radiation Oncology, Shandong Provincial Key Laboratory of Precision Oncology, Shandong Cancer Hospital and Institute, Shandong First Medical University and Shandong Academy of Medical Science, Jinan 250000, China; Department of Biostatistics, School of Public Health, Cheeloo College of Medicine, Shandong University, Jinan 250012, China; National Institute of Health Data Science of China, Shandong University, Jinan 250000, China; Department of Gastrointestinal Surgery, Shandong Cancer Hospital and Institute, Shandong First Medical University and Shandong Academy of Medical Science, Jinan 250000, China; Department of Biostatistics, School of Public Health, Cheeloo College of Medicine, Shandong University, Jinan 250012, China; National Institute of Health Data Science of China, Shandong University, Jinan 250000, China

## Abstract

**Motivation:**

Enzymatic reaction play a pivotal role in regulating cellular processes with a high degree of specificity to biological functions. When enzymatic reactions are disrupted by gene, protein, or metabolite dysfunctions in diseases, it becomes crucial to visualize the resulting perturbed enzymatic reaction-induced multi-omics network. Multi-omics network visualization aids in gaining a comprehensive understanding of the functionality and regulatory mechanisms within biological systems.

**Results:**

In this study, we designed PhenoMultiOmics, an enzymatic reaction-based multi-omics web server designed to explore the scope of the multi-omics network across various cancer types. We first curated the PhenoMultiOmics database, which enables the retrieval of cancer-gene–protein-metabolite relationships based on the enzymatic reactions. We then developed the MultiOmics network visualization module to depict the interplay between genes, proteins, and metabolites in response to specific cancer-related enzymatic reactions. The biomarker discovery module facilitates functional analysis through differential omic feature expression and pathway enrichment analysis. PhenoMultiOmics has been applied to analyze the transcriptomics data of gastric cancer and the metabolomics data of lung cancer, providing mechanistic insights into interrupted enzymatic reactions and the associated multi-omics network.

**Availability and implementation:**

PhenoMultiOmics is freely accessed at https://phenomultiomics.shinyapps.io/cancer/ with a user-friendly and interactive web interface.

## 1 Introduction

Systems biology leverages genomic, proteomic, and metabolomic technologies to unravel the etiology and mechanisms of diseases within intricate biological systems. Integrating diverse biological components, including genes, proteins, and metabolites, enriches our understanding of interconnected cellular activities and the abnormal metabolic states present in diseases (Yang 2020, Cantini *et al.* 2021, Zheng *et al.* 2022, Shutta *et al.* 2023). A fundamental aspect of a cell’s biological omics cascade consists of an extensive set of enzymatic reactions, which are composed of substrates, products, and enzymes, making them the simplest systems to investigate regulatory mechanisms in disease (Yang 2004, Chen *et al.* 2017, Lieu *et al.* 2020, Akıncılar *et al.* 2023).

The rapid advancement of omics techniques, such as transcriptomics, proteomics, and metabolomics, enables efficient and precise profiling at the genomic, proteomic, and metabolic levels. Efficiently integrating these molecular components is essential for constructing a comprehensive framework that elucidates the relationship between phenotype and cellular characteristics (Ma *et al.* 2015, He and Xie 2021). Notably, computational tools and web servers have emerged to facilitate the integration and visualization of genomic, proteomic, and metabolomic data for mechanistic investigation in cancer, Alzheimer’s disease, and cardiovascular diseases (CVD). Specifically, for multi-omics data integration and analysis, the network-based approaches combine statistical models and functional analysis to prioritize the omics features that are functionally related to the phenotypes. Current network-based multi-omics web servers deploy unsupervised learning methods such as matrix factorization, Bayesian networks, random walk, or statistical correlation-based approaches (Langfelder and Horvath 2008, Vaske *et al.* 2010, Rodrigues *et al.* 2018, Liany *et al.* 2020, Wen *et al.* 2021). For example, Zhou *et al.* have developed OmicsNet, a user-friendly web server that allows researchers to effortlessly create and visualize biological networks in a 3D space based on biological features such as genes, proteins, and metabolites (Zhou and Xia 2018). MiBioMics was designed to generate correlation maps among multi-omics features and extract relevant variables connecting omics layers to a trait of interest (Zoppi *et al.* 2021).

Many multi-omics analysis tools require the input dataset that includes multiple omics dataset, e.g. transcriptomics, proteomics, and metabolomics, followed by conducting statistical correlation for these multi-omics features (Kuo *et al.* 2013, Hernández-de-Diego *et al.* 2018, Zhou *et al.* 2021, Liu *et al.* 2022). However, for studies that only have one omics dataset such as metabolomics, current multi-omics tools often could not map the metabolites to proteins and genes automatically. Enzymatic reactions start with interactions between metabolites and proteins at the active site of enzymes. By including enzymatic reaction information, gene–protein regulation information, and gene–disease association information, the enzymatic reaction inferred multi-omics network could be reconstructed. Though many enzymatic reaction databases have been developed, the enzymatic reaction-based multi-omics network web application is still very rare.

In this study, we designed PhenoMultiOmics, an enzymatic reaction-inferred multi-omics database that integrates 5540 enzymatic reactions across 45 cancer types, using data from the Metabolic Atlas, BRENDA database, RHEA database, and EnzymeMap database (Schomburg 2004, Robinson *et al.* 2020, Wang *et al.* 2021, Bansal *et al.* 2022, Heid *et al.* 2023, Li *et al.* 2023). This integration produces 759 558 gene–protein–metabolite–disease associations. PhenoMultiOmics incorporates a statistical and function analysis module for conducting differential omic analysis and generating multi-omics networks associated with diseases like gastric cancer and lung cancer. PhenoMultiOmics is hosted using R Shiny web applications at https://phenomultiomics.shinyapps.io/cancer/, offering a user-friendly interactive interface. As such, PhenoMultiOmics promises to greatly facilitate the study of multi-omics analysis with enzymatic reaction information, extending our understanding of mechanistic processes in biology and diseases.

## 2 Materials and methods

### 2.1 Construction of PhenoMultiOmics database

To construct the enzymatic reaction inferred multi-omics database, we extracted the enzymatic reactions from multiple enzymatic reaction databases, including Metabolic Atlas, BRENDA database, RHEA database, and EnzymeMap database (Schomburg 2004, Wang *et al.* 2021, Bansal *et al.* 2022, Heid *et al.* 2023, Li *et al.* 2023). Each enzymatic reaction is denoted as a chemical reaction equation with reactants and products, including the enzymes and metabolites involved in the reaction. For metabolites and proteins that are involved in the same enzymatic reaction, it is regarded as a protein–metabolite association. To generate the connection between genes, proteins, metabolites, and diseases, we then mapped the enzymes/proteins to associated genes using UniProt Knowledgebase, followed by generating the gene–disease association information based on the DisGeNET database (McGarvey *et al.* 2019, Piñero *et al.* 2019, Bateman *et al.* 2021). Therefore, the PhenoMultiOmics database (PMODB) includes 5540 enzymatic reactions across 45 cancer types, which incorporate 759 558 gene–protein–metabolite–disease connections. PMODB also comprises essential information about diseases, genes, proteins, and metabolites with links to external databases. For genes and diseases, it includes the disease name and ID, gene name, gene symbol, and gene–disease association score. Regarding proteins, the database contains information such as protein name, enzyme classification (EC) number, UniProt ID, enzyme name, protein sequence length, and the corresponding gene name for the protein. As for metabolites, PMODB includes metabolite names, chemical formulas, SMILES notation, and associated metabolic pathways.

### 2.2 Visualization of multi-omics network

Based on the established PMODB, a multi-omics network is generated utilizing enzymatic reaction data involving genes, proteins, and metabolites. In this network, each gene, protein, or metabolite is represented as a node. The enzymatic reaction information is used to create edges between genes, proteins, and metabolites. Specifically, in enzymatic reactions, edges denote interactions between proteins (enzymes) and metabolites (substrates and products). In addition, for genes that regulate protein expression, connections are established between genes and proteins, as well as between genes and metabolites. Considering the bidirectional or reversible nature of enzymatic reactions, the network does not contain the direction of reactions, and edge weights are not assigned (Pogodaev *et al.* 2019).

### 2.3 Biomarker discovery module

The PhenoMultiOmics web server incorporates a biomarker discovery module for statistical and functional analysis to discover the key differentially expressed multi-omics biomarkers in case–control studies. For statistical analysis, the univariate and multivariate analyses were embedded. Since the PMODB was designed based on enzymatic reactions with gene, protein, and metabolite information, the biomarker discovery module accepts the preprocessed transcriptomics, proteomics, and metabolomics datasets acquired from case–control cohorts. Specifically, it requires the matrices of gene expression, proteomics, or metabolomics data as input. Each row of this matrix represents a quantitative abundance value of a sample, and each column corresponds to a feature. The univariate analysis performs statistical significance testing to detect the different expressed omics features between case and control groups. For statistical tests, Benjamini and Hochberg (BH) correction could be applied to adjust the *P*-value if necessary. Results are presented in tables and volcano plots, with default criteria for a significance level of *P*-value <0.05 and an absolute Log2 Fold Change (Log2FC) ≥ 1. Multivariate analysis includes principal component analysis (PCA) and partial least squares discriminant analysis (PLS-DA) that performs dimensionality reduction and feature selection. Functional analysis using omics data aims to understand the biological functions and pathways associated with the genes, proteins, or metabolites identified in an omics study. This analysis facilitates the interpretation of the biological significance of the results, uncovering the underlying mechanisms of diseases or phenotypes. Gene Ontology Enrichment Analysis (GOEA) and KEGG Metabolic Pathway Analysis are embedded on the web server. GOEA identifies which GO terms (biological processes, cellular components, and molecular functions) are overrepresented given a set of genes or proteins. KEGG Metabolic Pathway Analysis identifies the metabolic pathways that are significantly affected in a metabolomics dataset. For the enrichment analysis, enrichment factors and BH-corrected *P*-values are computed to identify significant enrichment pathways, displayed via bar and bubble charts. Finally, the enrichplot R package is used to visualize networks of individual genes and pathways.

### 2.4 Multi-omics data integration module

The multi-omics data integration module accepts input of preprocessed multi-omics datasets, including transcriptomics, proteomics, and metabolomics. The module first generates the visualization of the uploaded dataset for visual inspection by t-SNE and UMAP, which aims to observe the distribution of the abundance of genes, proteins, and metabolites. Users have options to select which type of omics dataset for subsequent biomarker discovery analysis. After selecting one or multiple omics datasets, the module will conduct normalization to unify the scale of different omics datasets. Then, the dimensionality reduction and visualization will be performed to further inspect the normalized and integrated multi-omics data.

### 2.5 RNA-seq transcriptomics data processing

Gastric adenocarcinomas exhibit considerable heterogeneity among patients, and the identification of these subtypes can offer insights into cancer progression mechanisms and personalized treatment possibilities. The RNA-seq transcriptomics data was sourced from the publicly accessible database PRJNA152559, GSE35809 (Pandi *et al.* 2014). The dataset includes genome-wide mRNA expression profiles of 70 primary gastric tumors from an Australian patient cohort, categorized into metabolic (15 samples), invasive (26 samples), and proliferative subtypes (29 samples).

### 2.6 Metabolomics data processing

Metabolomics data were extracted from the Metabolomics Workbench (https://www.metabolomicsworkbench.org/) with study ID ST001231. The dataset consists of 31 plasma samples from individuals with lung cancer (case group) and 35 plasma samples from individuals without lung cancer (control group). The data were acquired using untargeted metabolomics via UPLC-QE-MS experiments, resulting in a metabolite intensity matrix for all samples, which was used for subsequent statistical analysis.

### 2.7 Implementation in R

The PhenoMultiOmics web server is programmed in R (version: 4.4.1) and relies on packages provided by Bioconductor and CRAN. The multi-omics network is constructed using the visNetwork package, while the limma package facilitates statistical analysis. All visualizations, including figures and plots presented on the web server, are created with the ggplot2 package and are available for download (Yu *et al.* 2012, Ito and Murphy 2013, Ritchie *et al.* 2015).

### 2.8 Long-term sustainability of PhenoMultiOmics

To maintain the relevancy and effectiveness of PhenoMultiOmics, we have implemented a version control strategy. We optimized the pipeline that retrieves the enzymatic reaction automatically with APIs to access these databases, including Metabolic Atlas, BRENDA database, RHEA database, and EnzymeMap database. The gene-disease information, compound information, and protein information will be updated by accessing public databases, such as DisGeNET, HMDB, and UniProt database. For the biomarker discovery module, we are constantly maintaining the applicability of statistical analysis and function analysis packages, including univariate analysis, multivariate analysis, correlation analysis, and pathway enrichment analysis. These updates will be performed every three months. These codes and data have been updated and deposited on the GitHub repository. It should be noted that human expert checking is needed if there are unexpected changes between versions. Moreover, we commit to ensuring transparency and user clarity by comprehensively documenting each version’s update details, including the nature and timing of data changes, on the PhenoMultiOmics platform. This method not only secures data timeliness but also facilitates user access to a historical data archive for comparative and retrospective analyses.

## 3 Results

### 3.1 Framework of the PhenoMultiOmics web server

**Figure 1.**
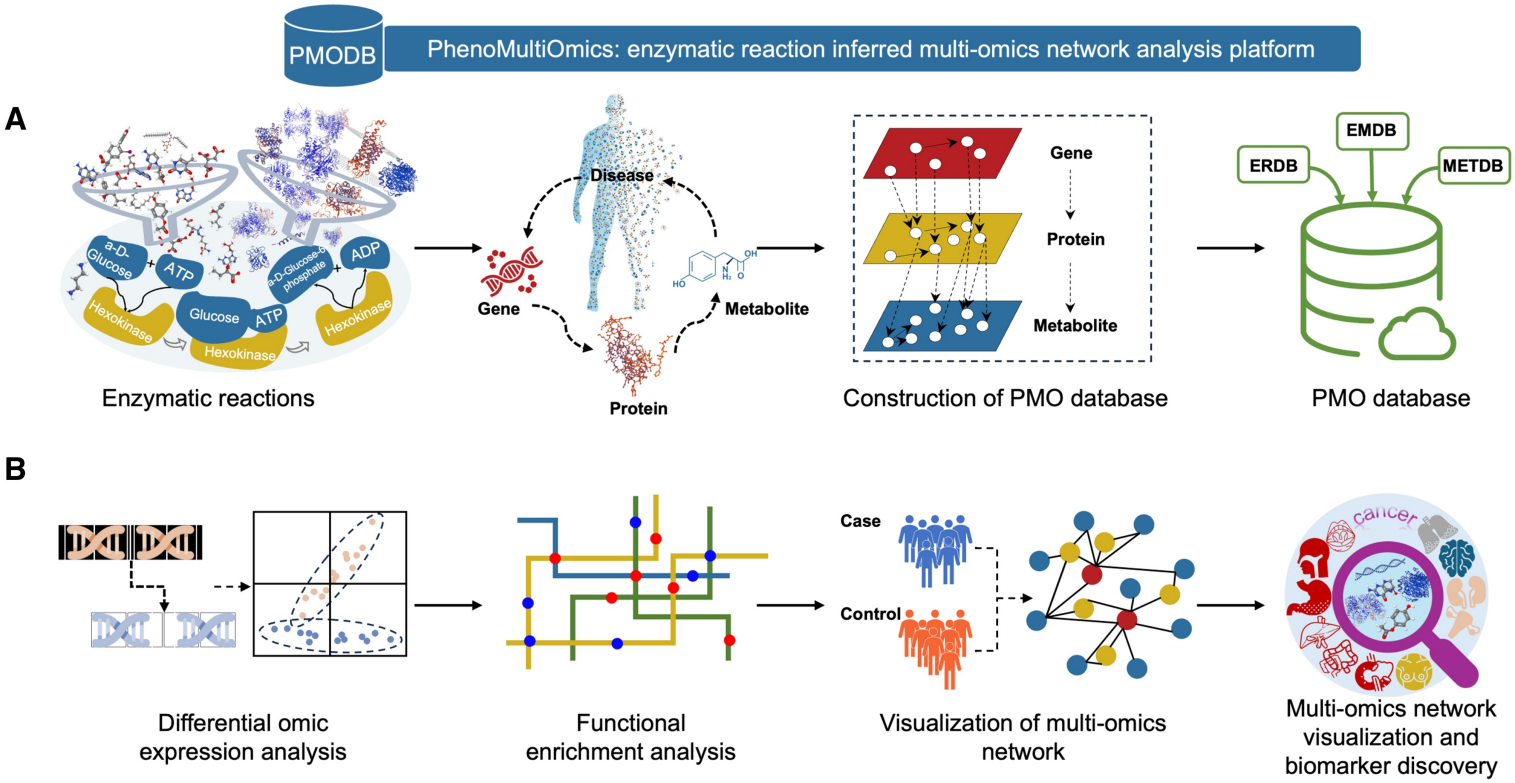
The overview of PhenoMultiOmics database and web server. Panel A shows the construction of PhenoMultiOmics database using thousands of enzymatic reactions. Panel B shows the biomarker discovery based on functional analysis and multi-omics network visualization, which includes differential omic expression analysis, functional enrichment analysis, and visualization of multi-omics network.

**Figure 2.**
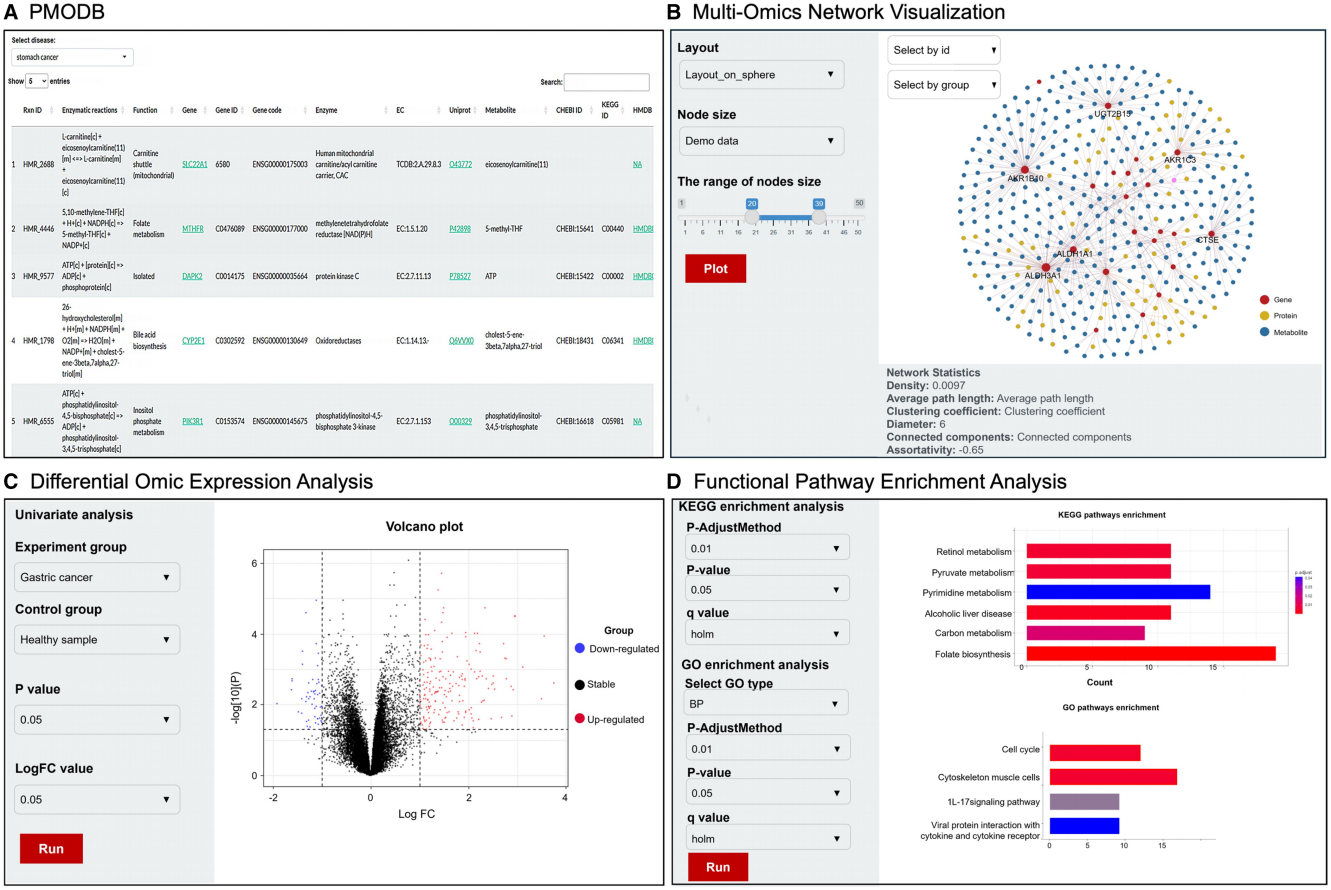
Screenshots of PhenoMultiOmics web server. Panel A shows the PMODB, which enables the retrieval of cancer-gene–protein-metabolite relationships based on the enzymatic reactions. Panel B shows the multi-omic network to perform visualization of reaction-based network information using disrupted genes, proteins, or metabolites in diseases. Panel C shows the differential omic expression analysis using demo data. Panel D shows the functional analysis using demo data.

The PhenoMultiOmics web server’s framework is illustrated in Fig. 1, featuring two key modules: PMODB, and PMO biomarker discovery. The PMODB is structured to store data records formatted according to enzymatic reactions involving genes, proteins, metabolites, and phenotypes. PMODB enables rapid access to multi-omics reaction relationship information by querying genes, proteins, or metabolites, with the query results available for download in tabular format. The PMO Biomarker discovery module encompasses differential omic analysis, gene set pathway enrichment analysis, and multi-omics network visualization facilitating the identification of disrupted genes, proteins, or metabolites and enabling functional analysis. Differential expression analysis results are illustrated using volcano plots, while enrichment results are displayed through bubble charts, column diagrams, and enrichment gene network diagrams for each pathway. The PMO multi-omics network, a visualization tool for reaction-based network information, highlights disruptions in genes, proteins, or metabolites related to diseases. In this network, each entity (gene, protein, or metabolite) is represented as a node, with connections based on enzymatic reactions as detailed in the Section 2. Figure 2 shows a screenshot of the PMO web server, demonstrating the capability to upload node and edge files for custom network diagram creation. Alternatively, users can upload only node files to generate multi-omics networks using the enzymatic reaction database. The interface allows users to customize the network layout and adjust the node size range according to their specific needs.

### 3.2 Application to gastric cancer transcriptomics data

**Figure 3.**
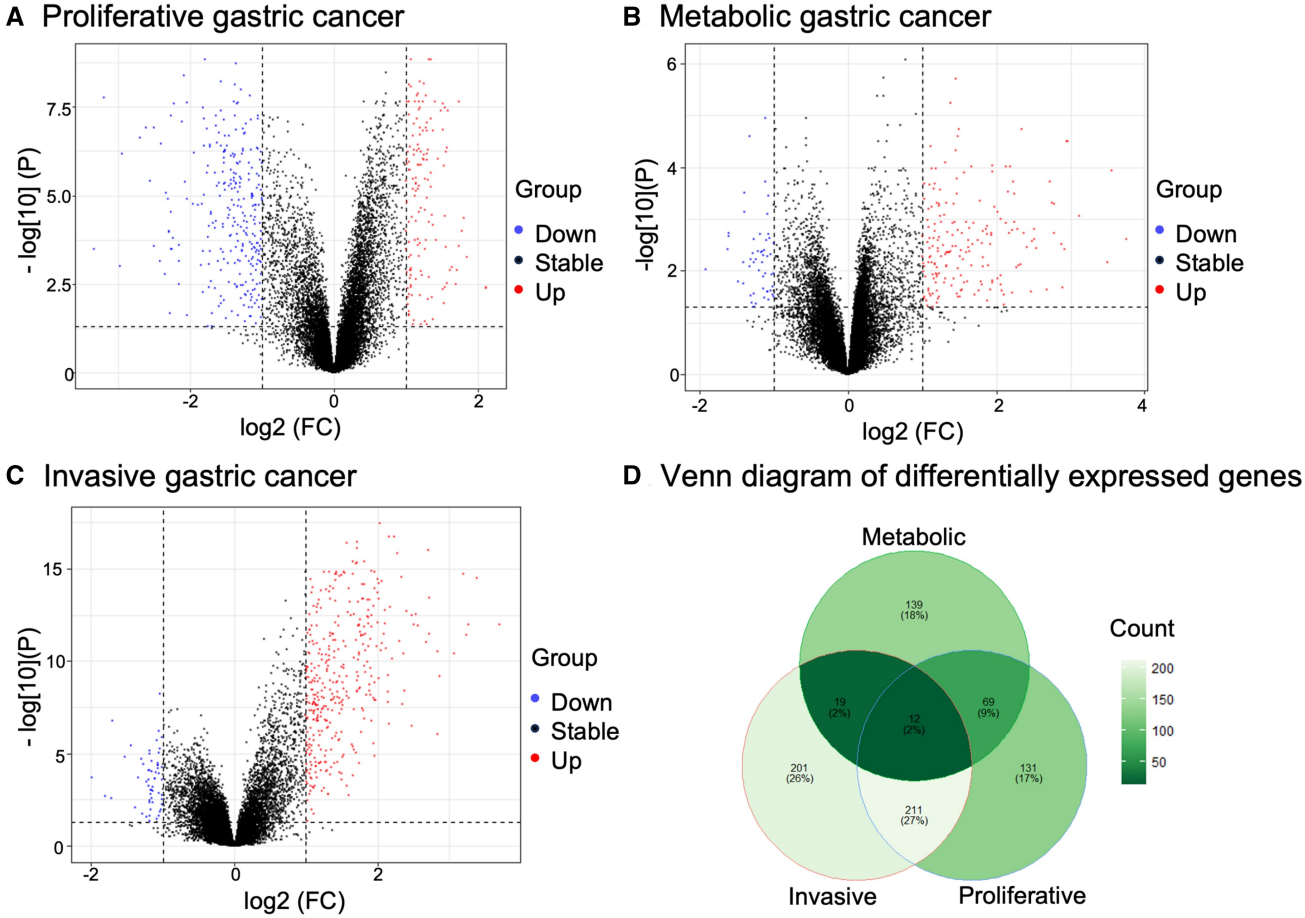
Volcano plots and Venn diagram of differentially expressed gene analysis in gastric cancer. Panels A, B, and C show results for different subtypes of gastric cancer. Panel D shows the Venn diagram of differentially expressed genes in three subtypes, which illustrates the common and distinct genes of three subtypes in gastric cancer.

Here we applied PhenoMultiOmics to analyze transcriptomics data from different subtypes of gastric cancer, including metabolic, invasive, and proliferative types. Figure 3 illustrates the outcomes of differential gene expression analysis, revealing significant differences in metabolic enrichment pathways among these distinct gastric cancer subtypes. Proliferative gastric cancer is notably associated with gene sets linked to the cell cycle (Kimura *et al.* 1992). Genome-scale metabolic reaction analysis reveals its enrichment in six pathways: Cell cycle, IL-17 signaling pathway, Viral protein interaction with cytokine and cytokine receptor, Progesterone-mediated oocyte maturation, Gastric acid secretion, and Motor proteins.

**Figure 4.**
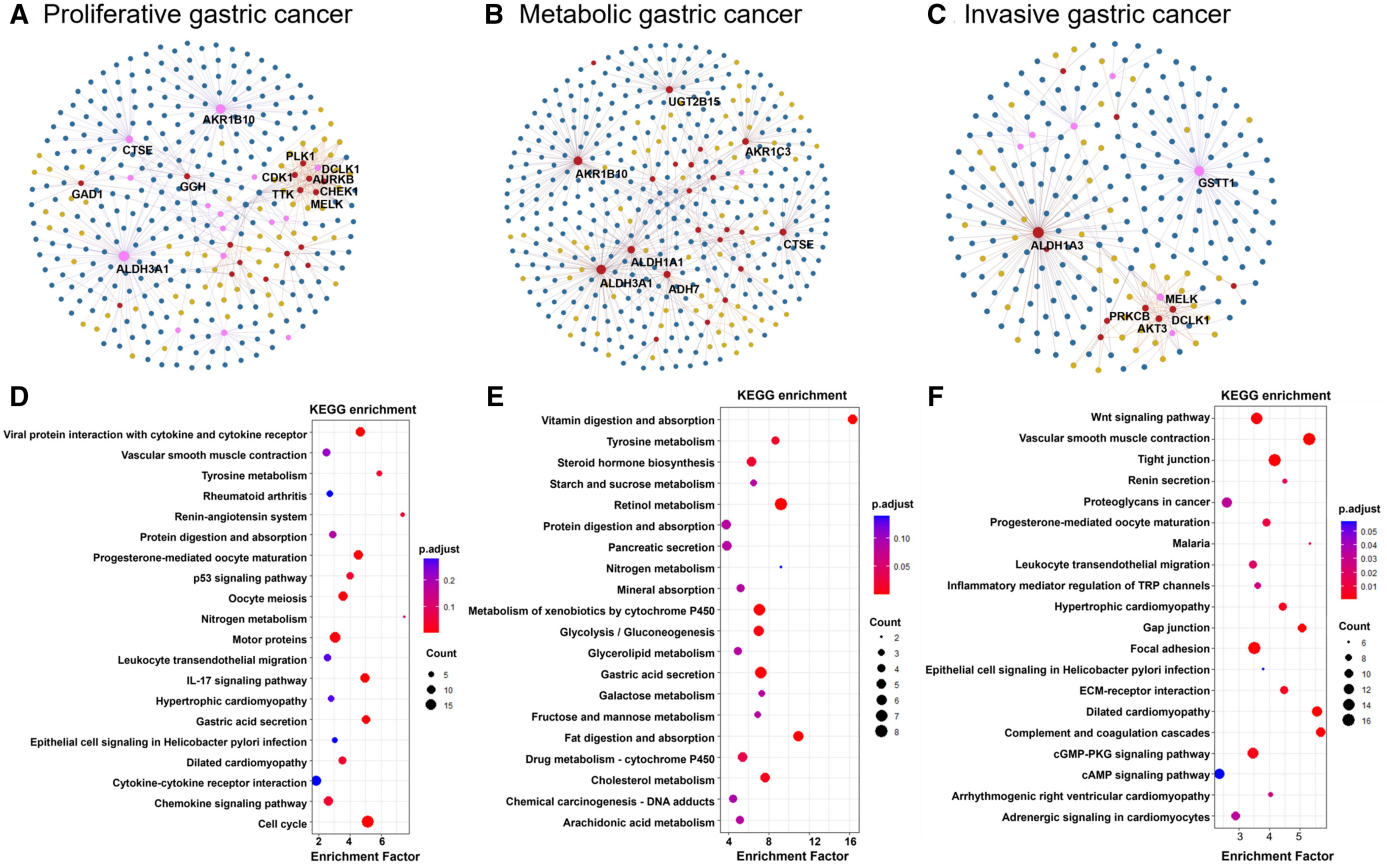
Multi-omics analysis based on differentially expressed genes in distinct subtypes of gastric cancer. Panels A–C show multi-omics networks based on enzyme reactions linked to differentially expressed genes in distinct subtypes of gastric cancer. Panels D–F depict the enrichment of metabolic pathways of differential genes for three subtypes of gastric cancer. Panels A and D denote the results of proliferative gastric cancer. Panels B and E denote the results of metabolic gastric cancer. Panels C and F denote the results of invasive gastric cancer.

PhenoMultiOmics multi-omics network analysis identified 250 enzymatic reactions, 41 genes, 64 proteins, and 148 metabolites related to gastric cancer. Mapping the differentially expressed genes of various subtypes onto this multi-omics network provided insights at the gene level for all three gastric cancer subtypes. As shown in Fig. 4, in these networks, TTK and CDK1 are prominent in modules linked to the Cell cycle and Progesterone-mediated oocyte maturation pathways, both critical for cell proliferation and motility in proliferative gastric cancer (Montazeri *et al.* 2021, Qi *et al.* 2021). By using gene ontology enrichment analysis, metabolic gastric cancer exhibits gene sets from several KEGG metabolism pathways and GO digestion, particularly enriched in pathways like Retinol metabolism, Metabolism of xenobiotics by cytochrome P450, Gastric acid secretion, Glycolysis, Gluconeogenesis, Fat digestion and absorption, and Vitamin digestion and absorption. Retinol and its metabolites play an important role in regulating cell proliferation and differentiation, and they also modulate immune responses (Larsson *et al.* 2007). Retinol can be irreversibly oxidized to retinoic acid, which is involved in regulating certain cellular functions such as cell growth, proliferation, and differentiation. For example, it can significantly inhibit the growth of human gastric cancer cell lines (Liu *et al.* 2001). Key nodes such as ALDH3A1 and ADH7 in the multi-omics network are notably enriched in Glycolysis/Gluconeogenesis and Metabolism of xenobiotics by cytochrome P450 pathways. The ADH7 gene is mainly expressed in the upper digestive tract and has been shown to be involved in the metabolism of exogenous substances through cytochrome P450 (Zhao *et al.* 2017). ALDH is an enzyme responsible for acetaldehyde oxidation, with reports indicating that cancer cells exhibit higher capability in ethanol oxidation but weaker ability in ethanol clearance (Jelski and Szmitkowski 2008). Besides their involvement in metabolizing exogenous substances through cytochrome P450 and drug metabolic pathways, ADH7, ALDH3A1, and ADH1B are also cross-genes with high connectivity within these pathways, indicating their potential use as diagnostic and prognostic biomarkers for gastric cancer (Zhao *et al.* 2017). Lei *et al.* (2022) identified the sensitivity of invasive gastric cancer to compounds targeting the phosphoinositide 3-kinase-AKT-mTOR pathway. Our enzyme reaction-based metabolic network analysis shows the PRKCB gene playing a significant role, enriching pathways like Vascular smooth muscle contraction, Focal adhesion, Gap junction, and the Wnt signaling pathway. Protein kinase C β (PRKCB) is a gene that encodes for the protein kinase C β protein, belonging to the protein kinase C family. PKC is a class of serine- and threonine-specific protein kinases that can be activated by calcium ions and the second messenger diacylglycerol. Members of the PKC family have distinct expression patterns and are considered particularly important in cancer-related processes (Dowling *et al.* 2016, Chen *et al.* 2019). Importantly, the Focal adhesion pathway is closely associated with Akt signaling and cell invasion.

### 3.3 Application to lung cancer metabolomics data

**Figure 5.**
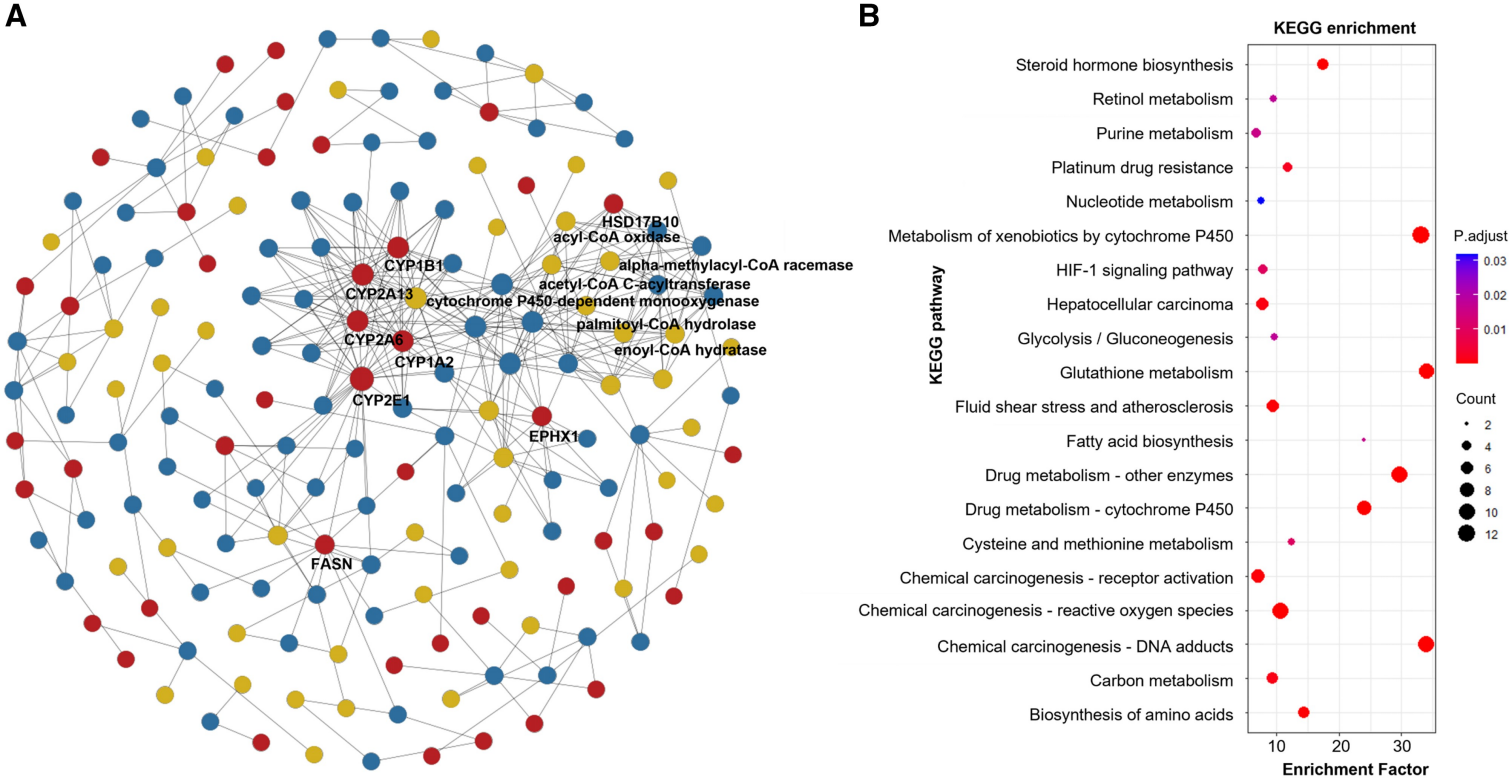
Multi-omics network visualization and functional analysis using disrupted metabolites of lung cancer. Panel A shows the results of the multi-omics network using the disrupted metabolites based on LC-MS metabolomics data of lung cancer. Panels B shows the results of metabolic pathway enrichment of differentially expressed metabolites in panel A.

Next, we extended the application of PhenoMultiOmics to the analysis of metabolomics data related to lung cancer. The differential omic expression analysis identified 3940 metabolic features. Within the multi-omics database, we identified 684 enzymatic reactions, 93 genes, 43 proteins, and 536 metabolites that constitute the multi-omics networks. Pathway enrichment analysis of these genes, as shown in Fig. 5, indicated their involvement in 20 distinct pathways. Within the multi-omics network, genes CYP2E1, CYP1B1, CYP1A2, and CYP2A6 exhibit close connections with the enzyme Unspecific monooxygenase. Notably, these genes are enriched in pathways such as Metabolism of xenobiotics by cytochrome P450, Chemical carcinogenesis—DNA adducts, and Drug metabolism—cytochrome P450. Polycyclic aromatic hydrocarbon (PAH)-derived active metabolites are important factors in the development of lung cancer. Stading *et al.* found that the formation of these active metabolites requires the metabolism of parent PAHs through cytochrome P4501A1/1B1 (CYP1A1/1B1) and epoxidase. These active metabolites then react with DNA to form DNA adducts, leading to mutations in key genes, such as the tumor suppressor gene p53, and are associated with lung carcinogenesis. PAH exposure also upregulates CYP1A1 transcription by binding to the aryl hydrocarbon receptor (AHR) and initiating transcription of the CYP1A1 promoter, which contains specific xenobiotic response elements (XREs) (Stading *et al.* 2021). Jia *et al.* found that CYP2E1 in the tissue surrounding tumors of patients with nonsmall cell lung cancer is significantly elevated. The novel CYP2E1 inhibitor Q11, namely 1-(4-methyl-5-thiazolyl) ethenone, is effective in treating lung cancer in mouse models. Furthermore, this study clarified that the benefits of Q11 might be related to the IL-6/STAT3 and MAPK/ERK pathways. These data suggest that CYP2E1 could be a new inflammation target and that Q11 exerts efficacy against lung cancer by modulating the inflammatory microenvironment. These findings provide a molecular basis for targeting CYP2E1 and demonstrate the feasibility of CYP2E1 inhibitor Q11 as a potential drug for lung cancer (Jia *et al.* 2023). A detailed examination of the Metabolism of xenobiotics by the cytochrome P450 pathway revealed the role of cytochrome P450-dependent monooxygenase in seven metabolic reaction pathways, highlighting its potential as a therapeutic target. Inhibiting their activity could disrupt the survival and proliferation of lung cancer cells, thereby enhancing the effectiveness of treatment. Lung cancer onset is closely linked to environmental factors like air pollution, tobacco smoke, and chemical exposures, which often include xenobiotics requiring metabolic processing. Cytochrome P450 enzymes play a pivotal role in handling these xenobiotics, as they convert certain chemicals into metabolites that are more readily excreted. Prolonged exposure to these environmental factors may elevate the risk of developing lung cancer (Dees *et al.* 1980, Elfaki *et al.* 2018). This underscores the scientific validity of extracting multi-omics information related to genes and proteins from the dimension of metabolites through a multi-omics network approach.

### 3.4 Limitations of the study

PhenoMultiOmics is curated based on existing enzymatic reactions that could elucidate complicated metabolic behavior and diverse cellular processes. There are a couple of limitations of PhenoMultiOmics. Since PhenoMultiOmics used enzymatic reactions to generate the connection between gene-protein-metabolite-disease, it is tailored specifically for pre-processed RNA-seq transcriptomics, proteomics, and metabolomics data analysis. Other omics datasets such as GWAS, epigenomics, and post-translational modification proteomics, will not be compatible with this tool. In addition, due to the limitation of computational expenses, for biomarker discovery module, it requires pre-processed datasets with genes, proteins, and metabolites. Therefore, preprocessing such as data imputation, standardization, and normalization should be performed prior to conducting the statistical and functional analysis. We have provided the detailed instructions how to conduct these preprocessing steps in the Tutorial section on the web server.

## 4 Conclusions

In this study, we have developed PhenoMultiOmics, a web-based tool anchored in enzymatic reactions, to integrate statistical and functional analysis for the swift analysis and visualization of multi-omics data. Demonstrating its utility, PhenoMultiOmics was applied to cancer transcriptomics and metabolomics datasets. This tool features a biomarker discovery module enabling differential omic analysis and the generation of disease-specific multi-omics networks, with case studies on gastric cancer and lung cancer. These case studies underscore the tool’s efficacy, showing a high level of concordance between PhenoMultiOmics results and the known biological metabolic reactions associated with these diseases. PhenoMultiOmics is poised to significantly enhance biomarker discovery and deepen our understanding of the intricate cellular activities and altered metabolic states prevalent in diseases.

## Supplementary Material

btae623_Supplementary_Data

## Data Availability

The web application is available at https://phenomultiomics.shinyapps.io/cancer/. The source code and processed data used in the manuscript is deposited on Github https://github.com/mmetalab/PhenoMultiOmics.
