# Supplementary material for: PhenoMultiOmics: an enzymatic reaction inferred multi-omics network visualization web server
Source: Bioinformatics. 2024 Oct 17;40(11):btae623. doi: 10.1093/bioinformatics/btae623 (PMC11549024; doi:10.1093/bioinformatics/btae623)
Supplement: btae623_Supplementary_Data [file btae623_supplementary_data.docx]

*Supporting information*

**PhenoMultiOmics: an enzymatic reaction inferred multi-omics network visualization web server**

Yuying Shi^1,2,3,†^, Botao Xu^4,†^, Zhe Wang^5,†^, Qitao Chen^1,2^, Jie Chai^4,*^, and Cheng Wang^1,2,^*

^1^ Department of Biostatistics, School of Public Health, Cheeloo College of Medicine, Shandong University, Jinan 250012, China

^2^ National Institute of Health Data Science of China, Shandong University, Jinan 250000, China

^3^ National Science Library (Chengdu), Chinese Academy of Sciences, Chengdu 610299, China

^4^ Department of Gastrointestinal Surgery, Shandong Cancer Hospital and Institute, Shandong First Medical University and Shandong Academy of Medical Science, Jinan 250000, China

^5^ Department of Radiation Oncology, Shandong Provincial Key Laboratory of Precision Oncology, Shandong Cancer Hospital and Institute, Shandong First Medical University and Shandong Academy of Medical Science, Jinan 250000, China

* To whom correspondence should be addressed.

Jie Chai

Email: [jchai@sdfmu.edu.cn](mailto:jchai@sdfmu.edu.cn)

Cheng Wang

Email: [chengwang@sdu.edu.cn](mailto:chengwang@sdu.edu.cn)

† The authors wish it to be known that, in their opinion, the first three authors should be regarded as Joint First Authors

**Comparison web server-based tools for multi-omics data analysis and visualization.**

The comparison of PhenoMultiOmics and other web-based multi-omics data analysis tools reveals significant differences and unique features across the platforms. Specifically designed for comprehensive cancer research, PhenoMultiOmics integrates transcriptomics, proteomics, and metabolomics data, leveraging enzymatic reaction databases including Metabolic Atlas, BRENDA database, RHEA database, and EnzymeMap database to inferred multi-omics network, as described in the Methods section. In contrast, other tools like MiBiOmics offer robust multivariate statistical tools such as Procrustes analysis and multiple co-inertia, which help in detecting and visualizing associations across different omics layers. MiBiOmics also features innovative visualization techniques like hive plots to summarize significant associations between omics-specific modules. MetaboAnalyst and 3Omics provide comprehensive analysis for transcriptomics and metabolomics, utilizing KEGG for pathway enrichment and differential analysis. These tools incorporate statistical correlation-based network analysis and topology analysis, enhancing their pathway enrichment capabilities. The comprehensive comparison of the key features in different platforms is provide in Table S1.

**Table S1.** Comparison of key features of PhenoMultiOmics with other web server-based tools for multi-omics data analysis and visualization.

| **Tools** | **Integration Capabilities** | **Database** | **Statistical**  **Analysis** | **Functional**  **Analysis** | **Multi-omics Network**  **Visualization** | **Source** |
| --- | --- | --- | --- | --- | --- | --- |
| PhenoMultiOmics | Transcriptomics, Proteomics, Metabolomics | Reactome, KEGG, Metabolic atlas | PCA, PLS-DA Differential analysis | Go enrichment,  Pathway enrichment | Enzymatic reaction-infered network analysis | <https://phenomultiomics.shinyapps.io/cancer/> |
| GraphOmics^1^ | Transcriptomics, Proteomics, Metabolomics | Reactome | PCA, Clustering, Differential analysis | Go enrichment | N/A | <https://graphomics.glasgowcompbio.org/> |
| MetaboAnalyst^2^ | Transcriptomics,  Metabolomics | KEGG | PCA, Differential analysis | Pathway enrichment, Topology analysis | Statistical correlation-based network analysis | <https://www.metaboanalyst.ca/> |
| PaintOmics 3^3^ | Transcriptomics, Proteomics,  Metabolomics DNase-seq  miRNA-seq | KEGG | Pathway enrichment (ORA)  Clustering of pathways | Pathway enrichment (ORA)  Clustering of pathways | Pathway-based network analysis | [www.paintomics.org](http://www.paintomics.org) |
| 3Omics^4^ | Transcriptomics, Proteomics, Metabolomics | KEGG, HumanCyc | Correlation analysis,  Coexpression profiles  Phenotype analysis | Pathway enrichment (ORA),  GO enrichment | Statistical correlation-based network analysis | <https://3omics.cmdm.tw/> |
| MiBiOmics^5^ | Transcriptomics, Proteomics, Microbiome | N/A | PCA, PCoA | N/A | Weighted Gene Correlation Network Analysis (WGCNA) | <https://shiny-bird.univ-nantes.fr/app/Mibiomics> |
| OmicsAnalyst^6^ | Transcriptomics, Proteomics, Metabolomics | KEGG | PCA, Differential analysis | Pathway enrichment | Statistical correlation-based network analysis | [^4^](https://www.omicsanalyst.ca/) |
| OmicsNet^7^ | Transcriptomics, Proteomics, Metabolomics  Microbiome | KEGG,  HMDB,  STRING | N/A | Pathway enrichment | Statistical correlation-based network analysis,  Database-inferred network analysis | <https://www.omicsnet.ca/> |
| ExpressVis^8^ | Transcriptomics, Proteomics | KEGG, GO | PCA, Clustering, Differential analysis,  Survival analysis | Pathway enrichment | Protein-protein interaction network | <https://omicsmining.ncpsb.org.cn/ExpressVis> |


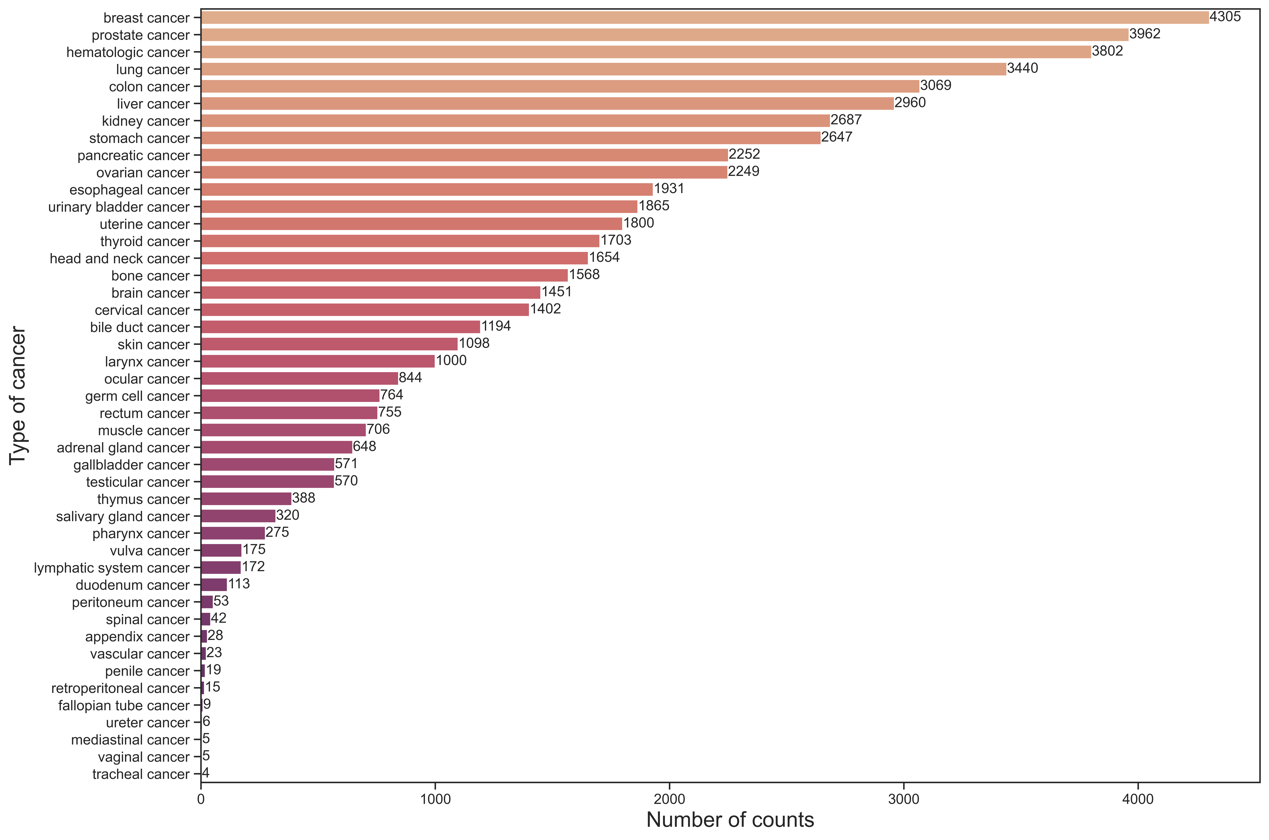


**Figure S1.** Bar plot of the number of enzymatic reactions in different cancer types in PMODB.


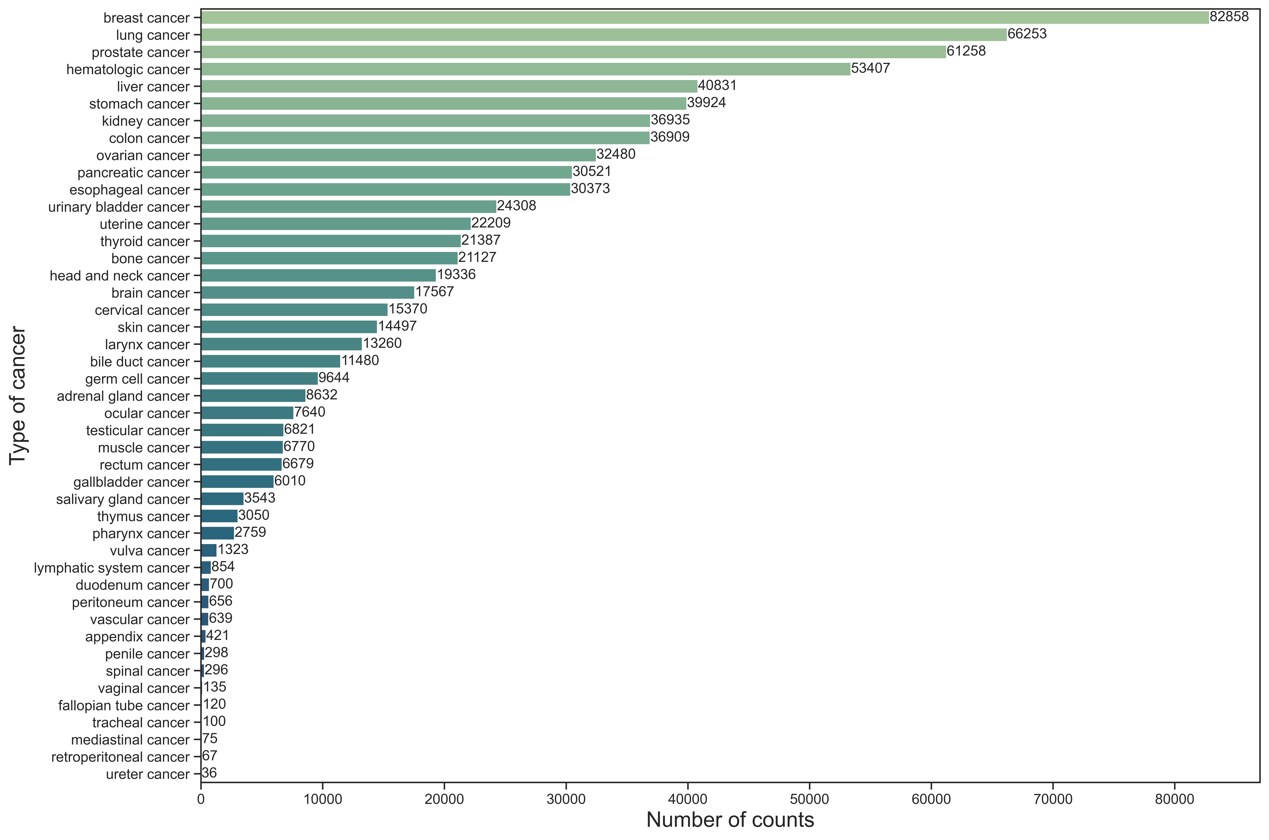


**Figure S2.** Bar plot of the number of gene-protein-metabolite associations in different cancer types in PMODB.


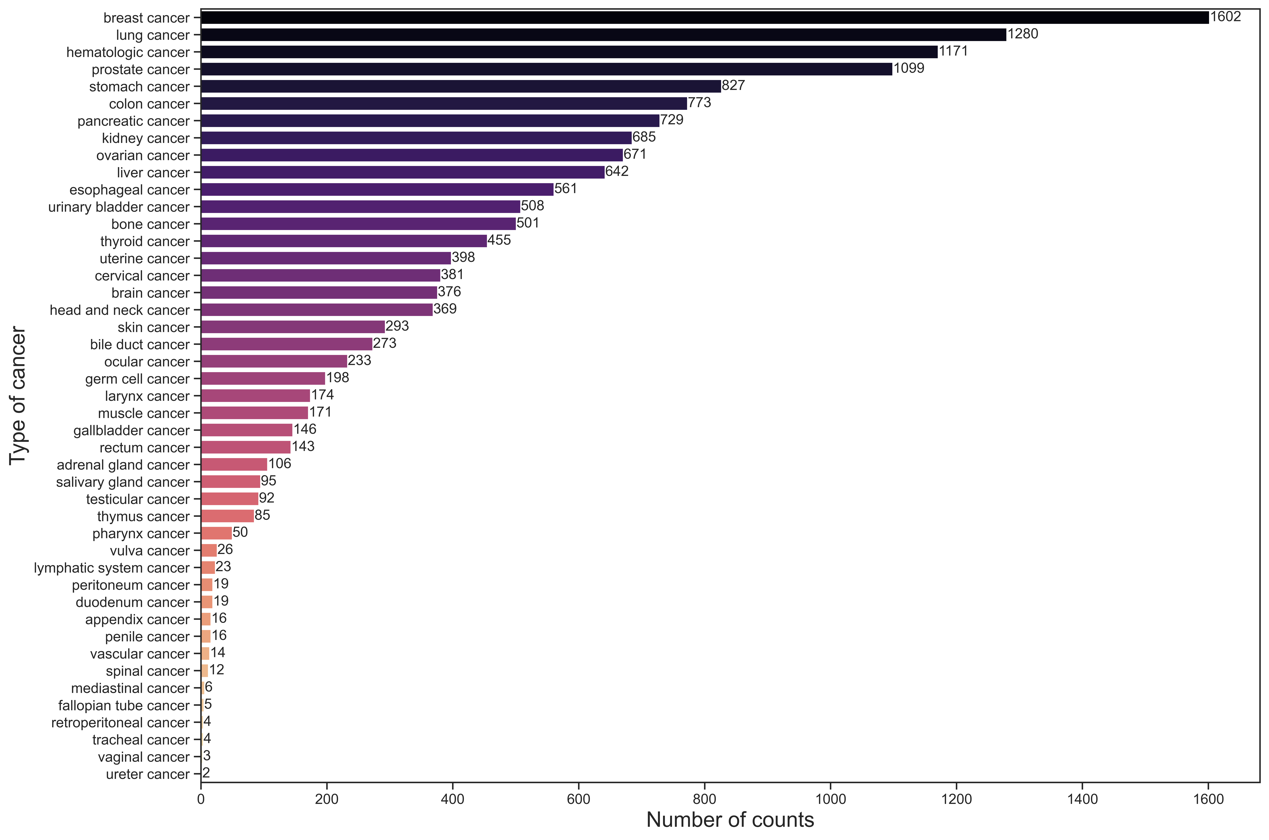


**Figure S3.** Bar plot of the number of genes in different cancer types in PMODB.


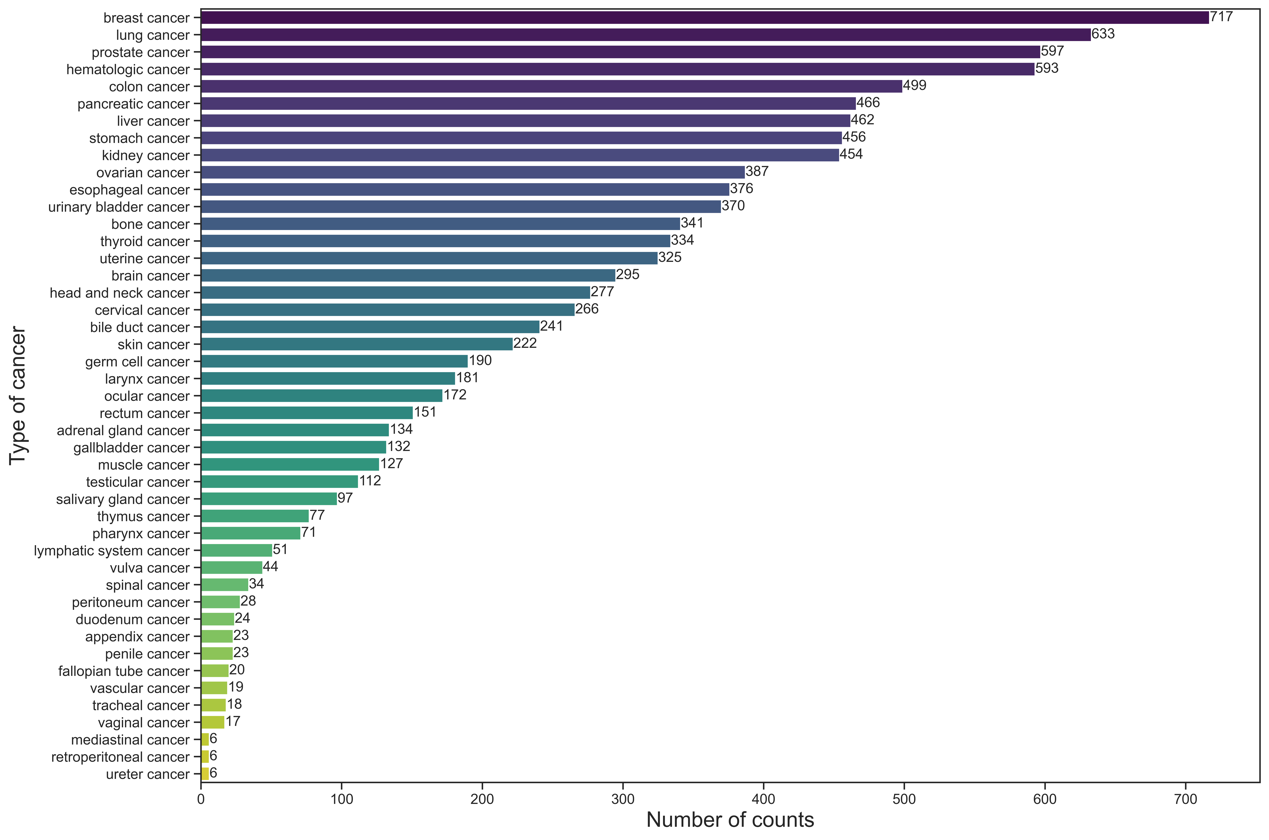


**Figure S4.** Bar plot of the number of proteins in different cancer types in PMODB.


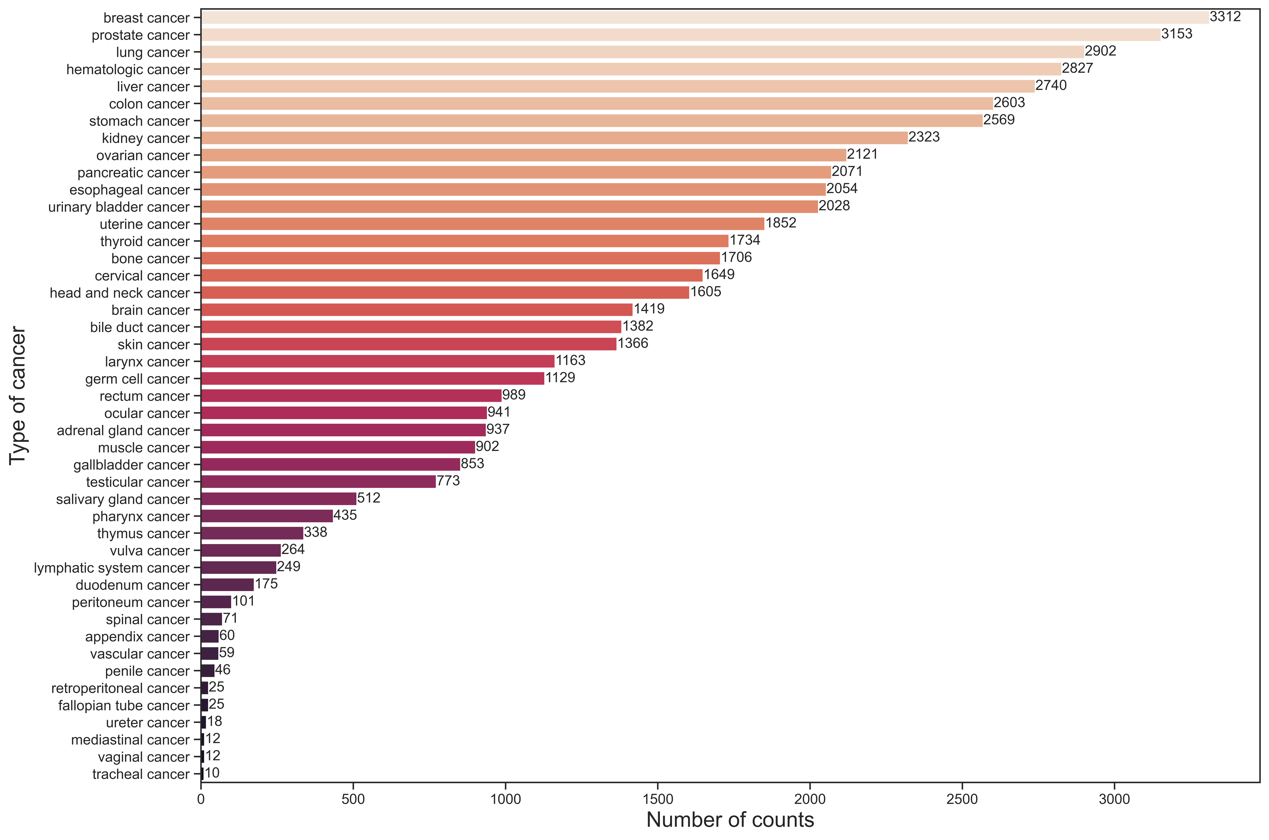


**Figure S5.** Bar plot of the number of metabolites in different cancer types in PMODB.


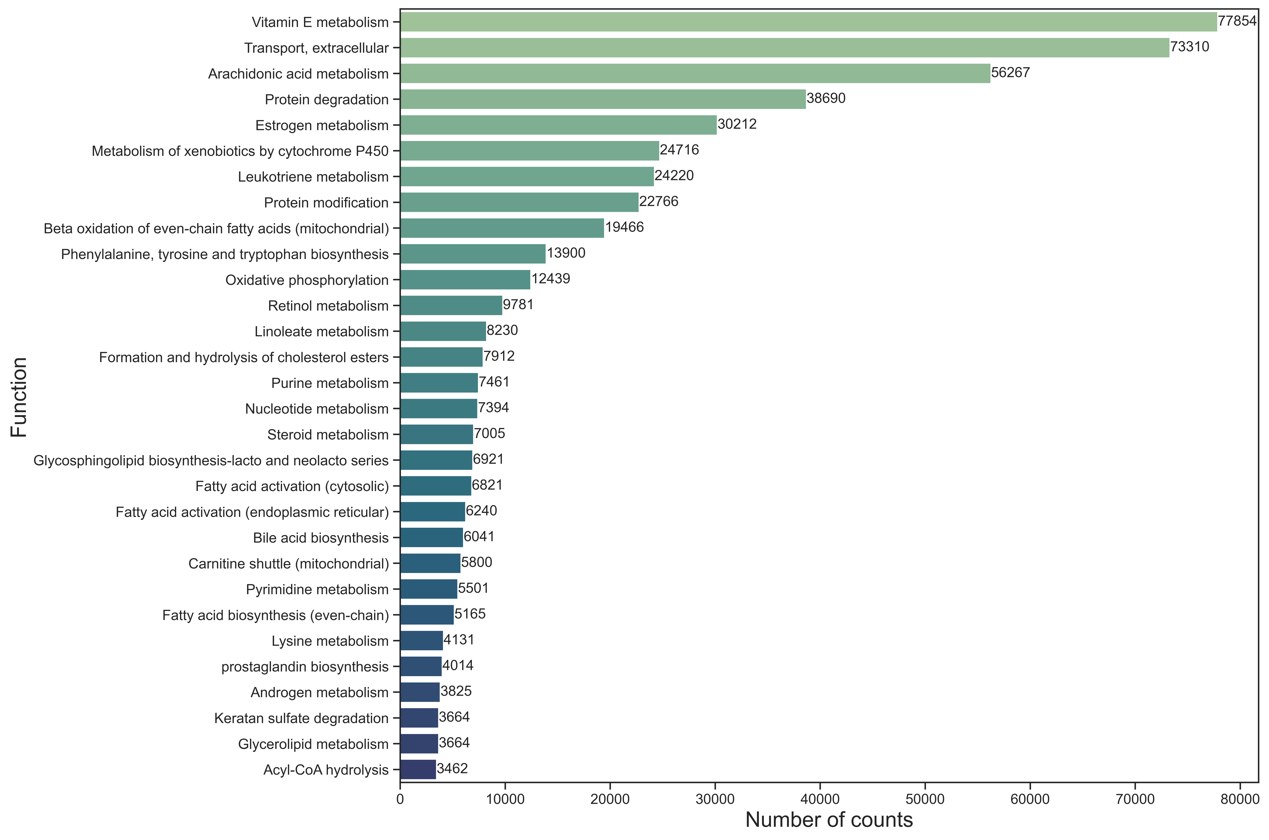


**Figure S6.** The top 30 most frequent functional systems in PMODB.

**References:**

1. Wandy, J. & Daly, R. GraphOmics: an interactive platform to explore and integrate multi-omics data. *BMC Bioinformatics* 22, 603 (2021).

2. Pang, Z. *et al.* MetaboAnalyst 6.0: towards a unified platform for metabolomics data processing, analysis and interpretation. *Nucleic Acids Research* 52, W398–W406 (2024).

3. Hernández-de-Diego, R. *et al.* PaintOmics 3: a web resource for the pathway analysis and visualization of multi-omics data. *Nucleic Acids Res* 46, W503–W509 (2018).

4. Kuo, T.-C., Tian, T.-F. & Tseng, Y. J. 3Omics: a web-based systems biology tool for analysis, integration and visualization of human transcriptomic, proteomic and metabolomic data. *BMC Syst Biol* 7, 64 (2013).

5. Zoppi, J., Guillaume, J.-F., Neunlist, M. & Chaffron, S. MiBiOmics: an interactive web application for multi-omics data exploration and integration. *BMC Bioinformatics* 22, 6 (2021).

6. Zhou, G., Ewald, J. & Xia, J. OmicsAnalyst: a comprehensive web-based platform for visual analytics of multi-omics data. *Nucleic Acids Research* 49, W476–W482 (2021).

7. Zhou, G., Pang, Z., Lu, Y., Ewald, J. & Xia, J. OmicsNet 2.0: a web-based platform for multi-omics integration and network visual analytics. *Nucleic Acids Research* 50, W527–W533 (2022).

8. Liu, X. *et al.* ExpressVis: a biologist-oriented interactive web server for exploring multi-omics data. *Nucleic Acids Research* 50, W312–W321 (2022).
